# Early usage and meaning of evolvability

**DOI:** 10.1002/ece3.5002

**Published:** 2019-03-11

**Authors:** Brian I. Crother, Christopher M. Murray

**Affiliations:** ^1^ Department of Biology Southeastern Louisiana University Hammond Louisiana; ^2^ Department of Biology Tennessee Technological University Cookeville Tennessee

**Keywords:** 1931, ability to evolve, John A. Thomson, Richard Dawkins, Sydney Fox

## Abstract

Evolvability has become an enormously popular concept in evolutionary biology and in machine learning software architecture. While it is claimed that the term was coined in 1988 by Richard Dawkins, it was used as early as 1931 as a characteristic of life by John A. Thomson. We quote and review the earliest uses and definitions of evolvability in biological frameworks up until 1989, which are remarkably few. The meaning changed from simply the “ability to evolve” as a characteristic of life to various versions of including necessary variation to predict whether or not something could evolve to the rate and quality of that evolution. Or, meaning changed from the ability to evolve to the “quality” of the ability to evolve. Since then, evolvability has taken on many definitions as it has exploded in usage.

Over the past twenty years, there has been increasing interest in ‘evolvability’ from within evolutionary biology and evo‐devo more specifically. (Brown, 2014: 550)

## 1 INTRODUCTION

Brown (2014) is a tour de force review and expansion of the concept of evolvability, but as implied in the quote above, Brown's exploration of the concept focused on fairly recent usage. No doubt, the past 20 years saw an explosion in the use of evolvability in biology and with that explosion of use came a myriad of definitions, which Brown does an excellent job of summarizing. For example, evolvability has been characterized as the ability to generate variation (Wagner, 2008; Wagner & Altenberg, 1996), the ability to exhibit phenotypic plasticity (West‐Eberhard, 2003), the capacity to reflect phenotypic variation from genotypic variation (Kirschner & Gerhart, 2006), the potential to generate exaptations (Maynard Smith & Szathmáry, 1995;), or just the standing genetic variation in a population (Houle, 1992). Ultimately, Brown (2014: 562) equated evolvability with “the broad disposition of populations to evolve” but noted that the various usages of the term were perhaps “proxies” for evolvability instead of some direct measure. All of these usages listed are similar, but subtly different, yet all involve either the capacity of populations to produce variation or simply the current variation present in a population at a given time, thus they all follow the Darwinian axiom that variation is required for evolution to occur, for example, “The capacity of populations to generate heritable phenotypic variation.” Since Brown (2014), the use of evolvability continued to subtly change. Crother and Murray (2018: 1) agreed with Brigandt (2015) and considered evolvability as a process that appears to “enhance/increase the production of variation/novel traits” as seen in a relative context. Koonin and Wolf (2016) even suggested that evolvability could be specific, biased, or even directed in a neo‐Lamarckian manner.

Pigliucci (2008), in a similar review of modern definitions of evolvability, highlighted how important the concept is to the direction of evolutionary biology, both theoretically and empirically. In this synthesis, Pigliucci (2008), in an attempt to overcome theoretical hurdles, suggested a consolidation of definitions (and implicated mechanisms; i.e., heritability, development, evolutionary transitions, etc.) into a family of overlapping ideas that require further refinement. Empirically, he noted the importance of quantifying the evolution of evolvability, that together with theoretical consideration, sets up the concept to be at the forefront of the extended evolutionary synthesis (EES), a contemporary revision of the Darwin‐based Modern Synthesis.

However, the purpose of this manuscript is not to review current thought on evolvability but to look at the earliest uses of the term and how it was conceptualized. Has the concept changed over time or is it essentially the same thing since its first uses? The goal of this essay is to report on the early history of the concept of evolvability and describe its use up to 1990.

## 2 METHODS

We used Google Scholar to quantify trends in the use of evolvability up to 1990. We stopped at 1990 because that is the decade where both Rosa (2017) and Brown (2014) begin their reviews. We searched the literature cited of relevant papers and followed threads. We read all the earliest uses and interpreted the meaning if the definition was not explicit in the publication.

## 3 RESULTS AND DISCUSSION

According to Sansom (2008), Dawkins (1988) coined evolvability. Dawkins may have been involved in popularizing and expanding on the concept, but he clearly did not enter it into the English lexicon. As far as we could determine, the earliest usage of evolvability was in 1931 (twice) by Sir J. Arthur Thomson (1931: 228, 231) in an essay titled “Biology and Human Progress” which was in the massive compendium *An Outline of Modern Knowledge* edited by William Rose. The volume is a collection of essays written by leaders in their respective fields that covered the current state of knowledge (at least western knowledge) in arts, sciences, history, business, psychology, philosophy, and more. The goal was to simplify the state of the knowledge so it would be accessible to a reader encountering the subjects for the first time. Thomson was a Scottish naturalist and widely known biologist, who although firmly held evolution to be the explanation for biodiversity, sought to reconcile science and religion and in the process popularized science. Perhaps, the need for distillation or simplification of a still young and complex topic, evolution, led Thomson to coin and use evolvability.

In “Biology and Human Progress,” Thomson used evolvability in the context of qualities organisms possess as living things. Thomson (1931:228) treated these qualities not as independent parts, but instead as integrated parts of a whole.

“Our point then, is that living creatures, which must be included in our philosophical picture of the world, show an integration of qualities. The fist triad includes:

1. Persistence amid incessant change;
2. The down‐breaking and up‐building of colloidal protoplasm; and
3. Specificity.

The second triad includes:

- Growth;
- Multiplication; and
- Development.

The third triad includes:

- Enregistration;
- Purposive behavior; and
- Evolvability.”

Three pages later Thomson (1931: 231) explicitly stated,

We have already mentioned evolvability as one of the nine characteristics of organisms…

The general claim that organisms are evolvable is difficult to reconcile with the necessity of variation for evolvable systems. Single organisms do not exhibit variation, but generations, populations, and species do. So we wonder if his usage of “organisms” being imbued with evolvability actually refers to the collective. His use of evolvability would certainly make more sense in the collective so we choose to interpret “organisms” as a collective, as populations and species of life, and not individuals.

Also in 1931, Thomson and Geddes published the two‐volume *Life: Outlines of General Biology*, which although the authors characterized as “our outline‐survey of Biology,” was truly a massive comprehensive study of biology with over 1,500 pages. Early on (p28), the authors wrote,

The facts and factors of organic evolution will be discussed in their proper place, here we are only concerned with pointing out that variability—and with it evolvability—must be ranked as one of the fundamental characteristics of living beings.

With regard to Thomson's previous description that evolvability is a characteristic of organisms, he switched to “living beings” which we interpret to mean life. This usage also diverged slightly from the former use where evolvability was recognized as a quality of organisms. Instead, Thomson and Geddes called Evolution the 9th quality and treat evolvability as a feature of evolution. To Thomson and Geddes (1931: 28), their evolution is Darwinian because of the necessity of variation, but also is not quite Darwinian, but a process imbued with need, “Whatever theory we hold as to the factors of organic evolution, we must leave room for the bent bow of endeavour.”

Regardless of the version of evolutionary theory held by Thomson and Geddes, the earliest usage of evolvability meant the ability (by whatever process) to evolve and it was used as a quality or characteristic to define living beings, life. While this general definition is the theme that runs through all the early usage of the word, numerous subtleties and variation in meaning emerge.

Thomson (1932) continued to use evolvability as a characteristic of life in the book *Scientific Riddles* (that is the title of the American edition, the London edition, also 1932, was titled *Riddles of Science*). In the former edition, the usage is markedly familiar (see Thomson and Geddes above). Thomson (1932: 28) wrote, “Finally, it must be recognized as characteristic of organisms that they give origin to what is new; they have evolved in the past, and the evolution of many is still going on. Variability and evolvability must be ranked as fundamental characteristics of living beings. Whatever theory is held in regard to the factors of organic evolution, room must be left for a large fact of life—the bent bow of endeavor.” The same issues about interpretation of “organisms” apply here as above. We find it significant that at the earliest usages, evolvability and variation are joined. However, Thomson leaves room for the interpretation that variation and evolvability are separate things and not necessarily inextricably joined. As a side note, Rosa (2017) in her review of evolvability considered Thomson (1932) the earliest usage of evolvability.

Woodruff (1941); Woodruff and Baitsell (1951) published seven editions of Foundations of Biology, which was considered a premier textbook at the time, “This book did more to unify the teaching content of introductory college courses throughout the country than had ever been done” (Nicholas, 1954) and starting in the 6th edition (1941: 598, 1951: 394) he used evolvability.

In the 6th and 7th editions (1941, 1951), in a chapter titled Origin of Species, he considered that phenotypic variation came from variation in germinal tissue and that these variants resulted from mutation. He tied germinal tissue variation with evolvability in this way, “The germ plasm never ceases to experiment, or natural selection to discover. Variability affording opportunity for adaptability is expressed in ‘evolvability’, a profoundly significant characteristic of life.” Interestingly, right after this passage Woodruff quotes Thomson, the apparent originator of the term evolvability, but the quote does not include evolvability. Regardless, it is clear that Woodruff read Thomson (even if Thomson was not cited in the references for that chapter) and so probably borrowed the term and the idea that it was an important characteristic of life. In addition, Woodruff expanded on the concept, to this characteristic of life, making it more than just the ability to evolve. Woodruff treated evolvability as a process to produce adaptations from the options among the available variation, which is not very different from some current views of the concept.

Woodruff's words reappear in the 1955 volume *Classics of Biology* (Suñer, 1955), which was a collection of some of the most important papers, or at least excerpt of papers, across the biological disciplines at that time. The quote from Woodruff is the same one noted above from *Foundations of Biology* with no embellishment by the editor.

To take a step backwards in time, the term evolvability was used by D. J. Cannon in 1950 in a paper titled “The Basic Concepts of Biology” and was published in the *Irish Journal of Medical Science*. It was 13‐page paper that, like Thomson and Woodruff, was a general review of biology. Cannon (1950: 451–452) wrote,

And this brings us to the most impressive characteristic of life, viz., its evolvability. Herbert Spencer seemed to think that the power to evolve is not a prerogative of living matter.

Cannon's meaning seems clear, evolvability is simply the ability to evolve and is again noted as a characteristic of life, but Cannon also appears to take issue with Spencer's view that other things, such as the cosmos in this case, could also evolve. The implication regarding Spencer's view is unclear, but if Cannon held Woodruff's view, it suggests Cannon did not think the cosmos evolved in the same manner as life, through the selection of variation, or because such variation did not exist in the cosmos. However, Cannon did not cite Woodruff, but cited J. Arthur Thompson [sic], so the complexity of Cannon's concept of evolvability may have been simple: life evolves. Unfortunately, Cannon was not explicit about the role of variation in evolvability. At least Cannon explicitly wrote “life” instead of “organisms.”

Evolvability does not appear again in the 1950s. From 1960 to 1969, the term appears seven times in the literature; however, four of those were about machine learning. Generally, these papers concern a machines ability to generate variation in the face of new information. One paper (Kabrisky, 1961: 1) on machine learning clearly tied the concept of evolvability to biological systems in a theoretical work in which a hypothetical model of the brain is the basis for machines being able to self modify, to learn on their own.

We interpret “iterable” as analogous to generations, thus descendant iterations could be different from the ancestor version and would be able to produce further variants in subsequent iterations. In this way, variation is a necessary aspect of evolvability in machine learning, as it is for life.

The year before the Nobel Laureate Hermann J. Muller retired, he published a paper about education in biology and in a discussion about the principles of life considered evolvability to be a characteristic of life, (Muller, 1963: 24):

All of them center about life's characteristic of *evolvability* (italics his).

Muller explained evolvability further,

This is a resultant of the unique combination of chemical faculties that the genetic material, in the form of chromosomes, that is, of chains of nucleotides, possesses. These faculties, all of which must have been simultaneously present to some degree in even the most primitive genetic material, may be listed as follows. There is, firstly, its faculty of replication—in other words, its tendency to unlimited expansion. Secondly, there is its susceptibility for undergoing varied chemical changes or mutations that are themselves replicable and that can be accumulated so as to attain an unlimited degree of elaboration. Thirdly, it is a prerequisite that this material, in some of its forms, should be effective in causing profound alterations in other materials around it, and that these alterations should be of diverse types, according to just what the constitution of the given genetic material is.

There seems little doubt that evolvability, as a characteristic of life, according to Muller, had to do with the generation of variation across generations, as did Kabrisky (1961, see above) with regard to machine learning. Considering Muller's research history on mutation and mutagenesis, it is not surprising he brought that into expanding the concept of evolvability. Where Woodruff certainly implied mutation as the ultimate source that drove evolvability, Muller was quite explicit.

In the popular news magazine *Science News Letter*, which was the precursor to the modern *Science News*, Wingo (1963: 147) wrote about Muller's view of what makes life in an article titled “Definition of Life.” Wingo was covering the American Institute of Biological Sciences meeting that year and quoted a number of workers about their definitions of life. Wingo lamented that most of the biologists resisted trying to define life and instead gave attributes of life. He said Muller claimed the basic characteristic of life is evolvability and added that Muller defined evolvability as “a unique combination of chemical faculties possessed by living things in the form of microscopic chromosomes.” We assume that these chemical faculties imbue life with the ability to generate variation and thus to evolve. As noted above, Wingo's description of Muller's evolvability is right in line with Muller's, 1963 writing.

Interestingly, the next use comes in a rebuttal to Muller's (1963) paper. Lammerts (1964: 44) took issue with Muller's claim that evolvability was a characteristic life, because, well, life did not evolve:

His discussions of the need for public understanding of the human genetics situation is very much to the point. However, I am at a loss to understand how Muller then follows up with his stress on teaching our boys and girls that the basic principles peculiar to the world of life center about life's characteristic of *evolvability* (italics his). Surely he must know that the most common characteristic of not only human populations but plant and animal populations as well is the accumulation of mutations which are defective.

Dr. Lammerts was the Director of Research in the Horticultural Research Division of the Germain Seed Company and so was no scientific novice, although he was apparently was a creationist, “In my opinion the two basic objectives in teaching biology or any other science are: (a) to impress the student with the glory of God our Creator as manifested by the marvelous complexity and design shown in nature….” Lammerts believed that if evolution through natural selection was correct, the adapted life forms should all be homozygous. The observation that species exhibit variation (which would be necessary for evolvability) ran counter to his belief that mutation could not be the source of such variation because mutation yielded defects and not traits that could be beneficial. Thus Lammerts (1964), through denial of the process of evolution, also treated evolvability as a characteristic of life that required variation via mutation.

Hanson (1977, 1981) authored two books on evolution and was a leader in biological education. While he was chairman of the national Commission on Undergraduate Education in the Biological Sciences, he (Hanson, 1966: 3) wrote a review essay “Evolution of the Cell from Primordial Living Systems” in which he discussed the evolution of cells from pre‐genic systems and pondered whether or not such pre‐genic systems had the capability to evolve, thus if evolvability was possible in primordial conditions,

One such approach is to ask, first, whether natural selection could act prior to the presence of a gene or gene‐like system…

In any case, the first question is the key one. Muller and Sagan tend to the view that pre‐genic natural selection, and therefore evolvability, is not possible. Oparin (1938; 1962) and others have argued in its favor. The prerequisites for evolution as we see it operating today are (1) the ability for self‐formation, (2) the ability to vary and to retain at least in some of the variants the self‐formative capacity, and (3) a finite environment which limits the number of living systems occupying given niches and thus forces them into competition.

The above two statements are tied together. The second quote refers to the first. Later, Hanson (1966: 3) added,

“We shall examine that viewpoint next, and in the process answer the question regarding evolvability as a property of a pregenic (sic) system.” From here, Hanson (1966: 4) eloquently made his case that pre‐genic systems could evolve.

The key problem is again evolvability through natural selection, for with the operation of natural selection there occurs an elimination of less successful forms and an accumulation of the more successful ones (Muller, 1929b). In the present context, natural selection cannot refer, of course, to changes in gene frequencies brought about through differential rates of reproduction, but rather to changes in amounts of the catalytic end‐products of different reflexive cycles as a result of different efficiencies of formation (Allen, 1957).

Hanson (1966: 5) realized he had more to solve, “As far as the question of evolvability is concerned, the prerequisites for evolution have already been outlined and it is the possibility of a ‘metabolically alive’ system functioning as a self‐formative, variable system that is problematical.”

Hanson (1966: 5) continued his arguments and concluded,

Thus we answer affirmatively the question whether or not evolvability is possible in pre‐genic system.

Hanson's use is interesting for two reasons, one because he explicitly recognized variation is key to evolvability and natural selection. He essentially said evolvability = variation + natural selection. The treatment of natural selection and evolution as synonyms may be a reflection of the state of thought at that time but his explicit recognition of the importance of variation for evolvability is similar to most ideas on the concept. The second interesting point, about non‐ or prebiological systems evolving is quite prescient when one considers the research program on pre‐genic systems of Sydney Fox, which is covered below. Overall, Hanson used evolvability in the same way as previous workers, that is, it is a characteristic of life, but one which prelife systems may also have possessed.

From 1970 to 1979, there were 47 references to evolvability, with eight about biological and prebiological systems while the remainder concerned machine learning. We include a single anthropological study on a specific aspect of Polynesian linguistics, the taxonomy and phylogeny of sibling terminology (Epling, Kirk, & Boyd, 1973). Essentially, the paper is about whether or not certain terminological partitions have the capability to evolve. Evolvability is used once (p 1600),

For the sake of generality, we can define ***evolvability* **(emphasis his) of a partition using face operators.

To explain that sentence, face operators are n‐cube graphs that can be broken into constituent binary pieces (partitions) in a step‐wise fashion. The partitions of the n‐cube represent terminologies and the partitions vary with regard to the number of variants that can be derived from any given partition or set of partitions. This brings the use of evolvability by Epling et al. (1973) into more familiar territory and allows us to interpret evolvability as the potential of partitions to yield variable forms. This definition seems to align more with evolvability as mutation rather than the *ability* to generate variation, but perhaps we are splitting hairs. Uniquely, Epling et al. bring potential into the concept, which presages later ideas (e.g., Dawkins, 1988), that is that some systems innately have more potential to produce variation and evolve than other systems.

The 1960s and 1970s witnessed a surge in interest and research on the origin of life and arguably, Sydney W. Fox was one of the leaders in this research in the 1970s. Fox was a well known and prolific scientist who pushed the envelope on hypothesizing the nature of prebiological structures that led to the origins of life. In the course of his work in the 1970s, he published at least three papers (Fox, 1973, 1974; Fox, Jungck, & Nakashima, 1974) in which he used evolvability. It is apparent in each of these examples that Fox used the term to mean ability to evolve.

In “Origin of the Cell: Experiments and Premises” Fox (1973: 8) wrote,

“While replicability is a prime component requirement for evolvability, communication between parent and offspring, and limited variability in synthesis of macromolecule are also required…” Here Fox argued that reproduction first appeared at the molecular level in simple pre‐genic structures, and he recognized that reproduction would be required for ancestor–descendant genealogies and concomitant descent with modification that would yield variation. With variation among the protocells, the protocells could now evolve.

A year later in “Coacervate droplets, proteinoid microspheres, and the genetic apparatus” he (Fox, 1974: 127) opined, “Since proteinoid, in appropriate systems, can synthesize internucleotide and peptide bonds, it provides the possibility of evolvability from a protocell.” The heading for the section which had the quote was “The Chicken‐Egg Questions” in reference to the debate over what came first in the origin of life, proteins, or nucleic acids. Fox argued that it was neither proteins nor nucleic acids, but instead proteinoids, or preproteins came first. Significantly for Fox, when these proteinoids interacted with water they spontaneously replicated, yielding the stuff of evolvability, that is variation.

Fox, with coauthors (Fox et al., 1974: 228), in a paper titled “From proteinoid microsphere to contemporary cell: formation of internucleotide and peptide bonds by proteinoid particles” noted that based on previous work (mostly Fox's) microspheres were “reproductive, evolvable, and heritable.” Because these pre‐cell structures could be involved in these processes, they stated Darwinian selection operated on them. The bulk of the paper sought to describe progress toward understanding the origin of protein and nucleic acid synthesis in a cell. In the final paragraph, Fox et al. (1974: 236) noted that although experimental evidence remained lacking for the evolution of a microsphere into a biological cell, those protocell microspheres could evolve, almost suggesting the development of a biological cell only would have been a matter of time,

The other context is the postulate that the proteinoid microsphere could evolve to a contemporary cell. This possibility has not been fully demonstrated, but evolvability of a model protocell has been demonstrated and the outlines of the total metamorphosis are clearer than they were.

The final session of the Leakey Foundation Symposium in 1973 was published in 1974 as *In Search of Man: Some Questions and Answers in African Archaeology and Primatology* (Campbell, 1974) and included the remarks made by the symposium speakers in the closing question and answer period. The participants were leaders and luminaries in their areas of research: Raymond Dart, Dian Fossy, David Hamburg, Richard Hay, F. Clark Howell, Glynn Isaac, Mary Leakey, and Jane van Lawick‐Goodall. Bernard Campbell chaired the session and asked the questions, one of which included the use of evolvability,

Dr. Campbell: Here's a question for Dr. Glynn Isaac. What is the spark of evolvability that permitted man to change so much, while his simian cousins remained basically the same?

In the response, Isaac did not in turn use evolvability, but provided an evolutionary context, “The modern understanding of evolution, right or wrong, is that it is a restless and opportunistic process in which animal numbers, in some sense, have a capacity to expand, and in which environmental considerations, a balance of species, restrains them. This creates a situation in which any change in the genetics of an animal population, which gives it an advantage, can lead to a trend in evolution.” It appears that Isaac interpreted evolvability to be a process dependent upon variation. Without the context of Isaac's answer, Campbell's use seemed to imply that evolvability is more than just a characteristic of life, but perhaps a variable trait or condition dependent upon another process to initiate it, that is the spark. Could Campbell have been thinking of a key innovation as the spark? If so, that certainly would depart from the typical thought of evolvability being dependent upon variation but could be considered as a constraint release that allowed the subsequent increase in variation.

In 1966, E. D. Hanson (see above) argued that pre‐genic systems exhibited evolvability. Eleven years later (Hanson, 1977: 5) in the volume *The Origin and Early Evolution of Animals*, he addressed organisms as units of evolution and wrote under the subheading *Organisms as evolvable systems,* “One answer to this problem is to recognize that the universal and unique property of living systems is their evolvability (Muller, 1955). One then asks, what is the biological function of this property? The answer is that evolvable systems are ones that take matter and energy from their environment to maintain and reproduce themselves and at least certain of their variants, and they do so in a limited environment…By accumulation of variations that allow the system to exploit more efficiently its environment, maintain and reproduce itself, we get the sequence of changes that constitutes evolution.”

So, Hanson treated evolvability as a characteristic of life, more specifically of an organism, and as the ability to evolve through the generation and selection of variation. This follows Thomson's ideas addressed above, which on the surface are peculiar in the sense that individual organisms themselves do not exhibit variation and hence, do not evolve. However, as we noted above, we interpret Thomson's “organisms” to not refer to individuals, but to the collective, life. The citation of Muller (1955) is interesting because nowhere in that paper does Muller mention evolvable or evolvability.

Liebau (1977) contributed a paper titled “Carapace ornamentation of the Ostracoda Cytheracea: principles of evolution and functional significance” to an edited volume on ecology and zoogeography of ostracods. In the abstract Liebau (1977: 107) wrote,

The components of fine sculpture of the Ostracoda are classified according to their variability and evolvability.

Liebau expounded on that (1977: 109),

The components of the fine sculpture, as far as studied in detail, are described here with special reference to their ‘evolvability’, i.e. how they can evolve. This ‘evolvability’ has been observed in phylogenetic lineages (list of genera). It has also been deduced from examples of intraspecific variation or constancy, respectively, which yielded general information on the ornament genetics.

The use of evolvability by Liebau is unique and even seems as if Liebau himself thought he invented the term, with the use of quotes around the two uses in the body of the text. If evolvability is “how they can evolve” it is absolutely intriguing that such an abstract concept could be employed in a systematics study. First, evolvability refers to traits as opposed to populations or lineages or clades. Perhaps, this use can be viewed as looking directly at the specific things that become variable: traits. In this sense, Liebau's meaning may not be far from those that emphasize variation at the population level. The deduction of how something evolves from the presence or absence of intraspecific variation may be foreshadowing the use of asymmetrically sized sister clades to hypothesize evolvability (e.g., Crother, White, & Johnson, 2007). Or, “how” can be interpreted as a process question regarding the specific traits. Liebau did mention the underlying genetics that may code for the trait, which would address the “how” question. Could Liebau's “how they evolve” question have referred to pattern (and not process) of character state change across ostracod phylogeny, therefore about transformation series? Evidence to support this interpretation comes from various descriptions of trait variation in different groups, with a good example in the following, “Together with the origin of the cythereidine intramural and mesh pores and with raised quantity of true marginal pore canals in advanced Hemicytherinae (Hemicytherini, Aurilini), there are four to five systems of pores which show a sudden increase in number of elements in the evolution of the Trachyleberididae. These phases are followed, at least in some branches of the family, by gradual reductions in the number of pores, a trend which corresponds with ‘Williston's law’ than the originating process.” The passage clearly noted pattern of change across phylogeny, contra to process. So, it seems to us that while process and pattern were both implied, we tend toward the pattern interpretation. Regardless, Liebau departed from a simple “ability to evolve” definition and added process and pattern, that is “how” to the meaning of the concept.

Between 1980 and 1989, Google Scholar found 154 papers that mentioned evolvability; however, only four were strictly with regard to biological systems. The remainder of uses are all in papers concerning machine learning. There was some crossover material, especially in the writings of Conrad (e.g., 1983, 1985, two of at least seven papers). His series of papers connect evolvability in biological systems with computer systems and for Conrad evolvability (the result of the presence of variation, or different pathways a thinking machine could pursue to solve a problem in more efficient ways) was an inherent trait of biological systems that was desired for machine learning systems. Conrad (1985: 676) puzzled over the lack of adaptability in computer systems and wondered over the solution to the problem, “The situation is summed up in a tradeoff principle: structural programmability is obtained at the cost of computational efficiency and evolutionary adaptability (Conrad, 1974a, 1984). Digital computers are built for programmability at the expense of efficiency and evolvability. But biological systems, as products of evolution, must have opted for evolutionary adaptability rather than for programmability. Evolutionary adaptability allows these systems to learn to use their computational resources efficiently. If all processes in nature are in principle simulatable by digital computers, it should be possible to simulate evolutionary processes. This can be done, but in order to do so it is necessary to pay the computational cost of simulating buffering mechanisms of the type which facilitate biological evolution.”

In the late 1970s and early 1980s, cultural anthropology was in the midst of a paradigm shift in which evolutionary explanations were being touted as critical to understanding cultural change over time (e.g., Adams et al., 1981). Previously, and currently at the time, functional ecological conclusions were being extrapolated into causal origin explanations. Some workers in the field recognized the seriousness of the problem that functional ecological studies could explain maintenance of cultural traits but evolutionary studies were required to address origin and change over time explanations. One of the leaders of the movement to push for evolutionary explanation was Paul Diener, and in an essay titled “On Distinguishing Functional Ecology and Evolution in Cultural Theory” he addressed the separation of operation (functional ecology) from origin (evolutionary) explanations in cultural anthropology. Diener (1980: 15) stated, “Rather, the distinction between functional ecology and evolution is absolutely vital to a proper understanding of the nature and origin of humanity. This conclusion is consistent with the biological literature, with what we know about complex system structures and processes, with recent reconsideration of the nature of “time” and “history” in evolving systems, and, indeed, with the tradition of theory within cultural anthropology itself. It is only in recent decades, under the influence of Steward and a mechanical materialism derived from him that the uniqueness of human “evolvability” (Muller, 1955: 3; Hanson, 1977: 5) has been questioned.”

The reference to Hanson (discussed above) suggests that Diener viewed evolvability similar to Hanson, and the context of Deiner's statement seems to support that. If Diener meant by “uniqueness of human evolvability” that natural selection on human variation was a unique process (perhaps because of their mental faculties?), then this fits well with our interpretation of Hanson (1966, 1977; see above). It is interesting that Diener cited both Muller (1955) and Hanson (1977) because Hanson also cited Muller (1955) but, as noted above, Muller (1955) did not use evolvability nor even evolvable in that paper. Perhaps Diener just followed Hanson's lead.

Braterman (1986: 152) contributed a brief essay titled “The Evolution of Evolvability: ‘The Pedigree Principle’” to a book on *Clay Minerals and the Origin of Life* (Cairns‐Smith & Hartman, 1986). The essay described a thought experiment in which clays could be argued as evolving systems, “What if our initial population varied in a rather more subtle and indirect way, namely in the accuracy with which its structure is copied into its own progeny? Call this property Q, varying between 0 and 1. Our first act of selection will be in respect of *P* only. Those selected members for which *Q = *0 will produce progeny with the full original spread of *P* and only a fraction of these will be re‐selected. Those selected members with high values of *Q* will successfully transmit *P* (and high *Q*.‐values) to their progeny with more than average efficiency, making sure that they are over‐represented in the next selection cycle. We are selecting for *P* explicitly, but for *Q* implicitly. Thus fidelity of replication will tend to improve, and hence the potential for evolution over the long term.”

Essentially, Braterman's thought experiment was a simplified description of the research program of Sydney Fox who previously claimed, and demonstrated, that pre‐cell pre‐genic systems could develop variation and evolve. While Braterman did not use evolvability in the paper, he apparently was the first to use “The Evolution of Evolvability” by predating Dawkins by one year. To get a better feel for what it means to be the first to use that title, for that combination of words, Google Scholar now finds about 29,400 instances. No doubt many of these are citations of the same papers, but one should get the picture. Braterman's paper garnered two citations, so the striking increase in usage speaks volumes about how that combination of words changed in significance.

In the volume titled *Artificial Life* (Langton, 1988), Dawkins (1988: 216) entered the discussion on evolvability with a chapter titled “The Evolution of Evolvability.”

Finally, let us return to the evolution of evolvability. The point I have been trying to make so far in this paper is that certain kinds of embryology find it difficult to generate certain kinds of biomorphs; other kinds of embryology find it easy to do so. It is clear that we have here a powerful analogy for something important about real biology, a major principle of real life that is illustrated by artificial life. It is less clear which of several possible principles it is!

Given the title of the chapter, one might expect more usage of the term evolvability, but the above quote is the only instance. The paper is a detailed description of the thinking Dawkins used for his computer program, “Blind Watchmaker,” in which phenotypes (biomorphs in the paper) can evolve. The above quote rediscovers an old idea and delivers irony to the use of evolvability and the relationship between machines/AI and biological systems. The recognition that some embryologies have more potential to evolve (generate variation) than other pathways is significant because it says that not only is there variation produced through evolvable systems, but that there is variation among the evolvable systems themselves in their ability to produce variation. If there is variation among evolvable systems, then evolvability itself would be under selection and Dawkins (2003) said just that. The embryologies that do not or have little potential to produce variation can be considered as developmental constraint (e.g., Maynard Smith et al., 1985; Raff, 1996; Crother et al., 2007). These views of constraint share the definition that they bias/hinder/inhibit/prevent the development and evolution of variable phenotypes. When any of these constraints are released, the result is an embryology with evolvability, that is new, novel phenotypes are able to emerge, the embryology regains the ability to evolve, which is what Dawkins discovered in his program. The irony comes from the fact that the early development of evolvability came from the machine learning/artificial intelligence research programs that looked to biological systems to inform them about developing machines that could evolve. Dawkins (1988) flipped that by noting that his artificial system is a “powerful analogy” that teaches us “something important about real biology.”

Wake and Roth (1989) edited a volume titled *Complex Organismal Functions: Integration and Evolution in Vertebrates* which was a report on a workshop held in Berlin in 1988. In that volume, Arnold et al. (1989: 410) wrote a group report on “How Do Complex Organisms Evolve?” In a discussion on the evolution of novelties they wrote,

Under the topic of ‘evolvability’ we discuss a special class of novelties, watershed events, that presage a proliferation of lineages and diversification in morphology.

It is clear that Arnold et al. (1989) shifted the definition of evolvability from a characteristic of life to a unique arena of evolutionary change, the evolution of key innovations (= “novelties, watershed events”) that lead to increased rates of speciation. Under the subtitle *The Evolution of Evolvability* (there's that combination of words again) Arnold et al. (1989: 412–413) concluded that evolvability was more than simply the ability to evolve, but in fact the ability to be “good at evolving.” Dawkins (1988) indicated the same thing, saying some embryologies are better at evolvability than other embryologies. In fact, we interpret “good at evolving” to cover both levels of evolvability as described above: the ability to generate variation and the ability of evolvable systems to evolve. Dawkins was a coauthor of the Arnold et al. paper so the resemblance of the ideas makes sense.

Lastly, Maynard Smith mentioned evolvability (1989: 242) in a paper titled “The Causes of Extinction” in a section subtitle: “*(a) Lack of evolvability, or running out of niche?*” It is the only time evolvability is used but the implication seems clear. Do populations go extinct because they cannot generate variation, therefore evolve, or because the products of mutation (variation) do not correspond to available niches? In this context, evolvability must mean besides ability to evolve but also perhaps, to borrow from Arnold et al. (1989), to be “good at evolving.” However, Maynard Smith suggested that regardless of the quality of evolvability, if the ecology presents no available options, then none of the generated variation will survive.

## 4 CONCLUSIONS

In summary, the utility and meaning of “evolvability” has itself varied through time and progressed to more modern definitions that encompass many past uses. Thomson (1931) followed by Woodruff (1941); Woodruff and Baitsell (1951) viewed evolvability as a characteristic of life and a feature of evolution in which endless experimentation produces variable opportunity to sustain existence. Such a sentiment was upheld independently by Cannon (1950) in rebuttal of Spencer's apparent over‐extrapolation of the term to nonliving cosmos. Further, consideration maintained the notion that evolvability referred to the generation of variation and that such generation was a characteristic of life, perhaps via mutation (Muller, 1963, reiterated by Wingo 1964) or pre‐genic processes (Fox, 1973, 1974; Hanson, 1966, 1977). The views of Muller (1963) specifically were refuted by the remnant creationist notions of Lammerts (1964), acknowledging that evolvability referred to the generation of variation via mutation; however, positing that this mechanism was not at play given the wand of a creator.

Anthropological discussion of human origin seemingly gave rise to convergent thinking about evolvability. Epling et al. (1973) discussed the division of languages, and the subsequent variation that such divisions result in, as the currency of language evolution. Testaments like this were expanded on by Campbell (1974; via Isaac symposia discussion) noting that human evolution was dependent on variation, and this ability to produce variation functioned like a trait subject to constraints. Discussion of human cultural origins also spurred a tautology in the discussion of evolvability as Diener (1980) reverted back to Hanson's sentiments and a thought pattern mirrored outside of anthropology by Braterman, 1986), who reiterated Fox's (1974) thoughts on nonliving variation restricting potential future variation. Just prior to modern considerations (reviewed by Pigliucci, 2008; Brown, 2014, Rosa, 2017), Arnold et al. (1989) and Maynard Smith (1989) formally acknowledged the inherent variation in evolutionary potential (hence relative evolvability) and the imposition of extrinsic constraints, respectively.

A further parallel, discussed above, is the concept of evolvability in machine learning. Kabrisky (1961) and Conrad (1985) used evolvability in the context of algorithmic self‐modifications and the efficiency provided by such variation, respectively. Dawkins (1988), while borrowing from biological thinking, intended to apply machine learning thought exercises to expand evolutionary considerations. Dawkins (1988) posited that variation is produced in evolvable systems and that production of variation in evolvable systems was, itself, variable, potentially hinting at the oppressive role of extrinsic factors on potential variability (i.e., constraints).

As illustrated above, the term *evolvability* has received sporadic yet parallel progressive attention in the 87 years of use reviewed above. Aside from machine learning language, the term most commonly refers to, with little dictated specificity other than as a characteristic of life, the ability (sometimes relatively) to evolve. This enigmatic use was inaccessible to quantitative evolutionary biology until the formalization of *evolvability* under a probability framework with variables reflecting intrinsic and extrinsic factors that weigh on possible extents of modification through time (Brown, 2014). This probabilistic framework allows the biologist to grasp relative probabilities of modification for specific traits or ecological influences in biotic systems. As illustrated by Crother and Murray (2018), specific developmental mechanisms applied to statements of evolvability allow for quantifiable relationships in evolutionary “ability” among clades, all else held constant.

From a theoretical perspective, implied numerous times before 1990, variation is required for evolvability, and there is variation in evolvability itself among clades because of relative degrees of constraint. Whether manifested in previously inherited form, mechanistic function, or external forces, traits are restricted to varying degrees of heritable change. The use of the term *evolvability* has been used with less attention to historical references than other biological terms, and definitions have repeatedly converged on a recently more formalized concept. While evolutionary biology has relied on quantifiable variation in the context of history, formal *evolvability* presents a novel chance to diagnose potential variation as well as its source, giving life to a predictive look at descent with relative modification.

When and why transitions in the meaning of *evolvability* occurred is a question to be answered; however, noteworthy patterns emerge from the discussion of *evolvability* presented above. Numerous authors present *evolvability* as a process‐oriented characteristic of life, as opposed to a pattern (Campbell, 1974; Cannon, 1950; Fox, 1973, 1974; Hanson, 1966, 1977; Maynard Smith, 1989; Muller, 1963; Thomson, 1931; Woodruff (1941); Woodruff and Baitsell (1951). One notable deviation from *evolvability*‐as‐process was Liebau (1977) in which relative evolvability was utilized as a trait of which the evolution was inferred using an Ostracod phylogeny. Along this path, Dawkins (1988) also implied that while the production of variation in evolvable systems is a process, the variation in such production may be viewed as a pattern.

Mechanistically, multiple biological phenomena have been implicated as both the drivers and/or sideboards of evolvability as a process. Muller (1963) explicitly identifies mutation as the driving force of variation and thus evolvability, whereas, Dawkins (1988) relates evolutionary potential to embryology, implying a developmental direction in his thinking. Seemingly, biological drivers of evolutionary potential were consolidated by Arnold et al. (1989) and Maynard Smith (1989) as things that produce variation within the boundaries of intrinsic and extrinsic constraints (perhaps thought to be a driver of relative evolutionary *potential* as opposed to variation). Other interpretations described above aim not to identify hypothesized drivers. Regardless of these hypotheses, the original meaning of evolvability was “the ability to evolve” and its earliest usages all concerned it being a characteristic of life. Along the way, evolvability became inherently incorporated as a characteristic of life because it is no longer touted as such and focus drew to mechanistic and process‐versus‐pattern hypotheses. Nevertheless, as Pigliucci (2008) elucidated, *evolvability* as a concept heavily contributes to a holistic sequel to Darwin's ideas, and indeed exemplifies an exciting advancement in evolutionary biology.

## CONFLICT OF INTERESTS

None declared.

## AUTHOR CONTRIBUTIONS

The study was conceived by BIC and CMM. The data were collected by BIC and CMM. All interpretations were done by BIC and CMM. The manuscript was written by BIC and CMM.

## Data Availability

No novel data were collected for this work. All the data are available in the cited works.
